# Audiovisual integration of the dynamic hand-held tool at different stimulus intensities in aging

**DOI:** 10.3389/fnhum.2022.968987

**Published:** 2022-12-14

**Authors:** Weiping Yang, Xiangfu Yang, Ao Guo, Shengnan Li, Zimo Li, Jinfei Lin, Yanna Ren, Jiajia Yang, Jinglong Wu, Zhilin Zhang

**Affiliations:** ^1^Department of Psychology, Faculty of Education, Hubei University, Wuhan, China; ^2^Brain and Cognition Research Center (BCRC), Faculty of Education, Hubei University, Wuhan, China; ^3^Department of Psychology, College of Humanities and Management, Guizhou University of Traditional Chinese Medicine, Guiyang, China; ^4^Applied Brain Science Lab, Faculty of Interdisciplinary Science and Engineering in Health Systems, Okayama University, Okayama, Japan; ^5^Research Center for Medical Artificial Intelligence, Shenzhen Institute of Advanced Technology, Chinese Academy of Sciences, Shenzhen, Guangdong, China

**Keywords:** audiovisual integration, stimulus intensity, older adults, dynamic hand-held tool, ERPs

## Abstract

**Introduction:** In comparison to the audiovisual integration of younger adults, the same process appears more complex and unstable in older adults. Previous research has found that stimulus intensity is one of the most important factors influencing audiovisual integration.

**Methods:** The present study compared differences in audiovisual integration between older and younger adults using dynamic hand-held tool stimuli, such as holding a hammer hitting the floor. Meanwhile, the effects of stimulus intensity on audiovisual integration were compared. The intensity of the visual and auditory stimuli was regulated by modulating the contrast level and sound pressure level.

**Results:** Behavioral results showed that both older and younger adults responded faster and with higher hit rates to audiovisual stimuli than to visual and auditory stimuli. Further results of event-related potentials (ERPs) revealed that during the early stage of 60–100 ms, in the low-intensity condition, audiovisual integration of the anterior brain region was greater in older adults than in younger adults; however, in the high-intensity condition, audiovisual integration of the right hemisphere region was greater in younger adults than in older adults. Moreover, audiovisual integration was greater in the low-intensity condition than in the high-intensity condition in older adults during the 60–100 ms, 120–160 ms, and 220–260 ms periods, showing inverse effectiveness. However, there was no difference in the audiovisual integration of younger adults across different intensity conditions.

**Discussion:** The results suggested that there was an age-related dissociation between high- and low-intensity conditions with audiovisual integration of the dynamic hand-held tool stimulus. Older adults showed greater audiovisual integration in the lower intensity condition, which may be due to the activation of compensatory mechanisms.

## Introduction

With the extension of life expectancy, the number of older adults continues to grow. Aging impairs sensory abilities, which makes it difficult for older adults to perceive stimuli in their surroundings (Chou et al., 2016; Jayakody et al., 2018). However, the effective use of multisensory information can help people better perceive external stimuli. Compared with single-sensory information, multisensory information can improve the probability and speed of the correct recognition of stimuli (Diederich and Colonius, 2004; Mercier and Cappe, 2020). The visual and auditory modalities are two important sources to obtain information from the outside world. The process of combining visual and auditory signals into a unified and stable percept is called audiovisual integration (Fendrich, 1993; Stein and Stanford, 2008). In comparison to the audiovisual integration of younger adults, the same process appears more complex and unstable in older adults (de Dieuleveult et al., 2017; Jones and Noppeney, 2021).

Many studies have compared audiovisual integration between older and younger adults under different stimulus types. Peiffer et al. (2007) used light emitting diodes and white noise as stimuli and asked participants to complete an audiovisual detection task. The results showed that under visual and auditory stimuli, there was no difference in the response time between the older adults and the younger adults and that under the audiovisual stimulus, the older participants responded faster than the younger participants. Then, the race model further found greater audiovisual integration in older adults than in younger adults (Peiffer et al., 2007). Later, researchers used event-related potential (ERP) technology to explore the facilitation of audiovisual speech information in older adults. The results showed that the amplitudes of P1 and N1 evoked by the audiovisual stimulus in the older adults were smaller than the sum of the amplitudes evoked by visual and auditory stimuli, and the observed P1 amplitude reduction was larger in older adults than younger adults. The reduced amplitudes of these components suggested that older adults could perform behavioral tasks well with fewer neural resources when audiovisual stimuli were presented simultaneously (Winneke and Phillips, 2011). Further research using magnetoencephalography (MEG) showed that both younger and older adults presented increased brain activity in response to audiovisual stimuli compared to visual or auditory stimuli presented alone after 100 ms stimulus onset. However, only older adults preferentially responded to audiovisual stimuli in the posterior parietal and medial prefrontal regions between 150 and 300 ms (Diaconescu et al., 2013). The results support the view that although the sensory processing ability decreases with age, audiovisual integration may provide a strategy that can ameliorate sensory deficits caused by a decline in single sensory processing and that brain activation is greater or more widespread in older adults than in younger adults, which might reflect compensation mechanisms with aging (Laurienti et al., 2006; Winneke and Phillips, 2011; Cabeza et al., 2018). Previous studies on audiovisual integration in older adults mostly used static simple meaningless stimuli or dynamic complex speech stimuli. However, few studies have explored the impact of dynamic hand-held tools on audiovisual integration in older adults. The complexity of dynamic hand-held tools, such as holding a hammer hitting the floor, is between static simple stimuli and dynamic speech stimuli, including the simultaneous presentation of object motion and body parts, which could reflect the motion-related process (Stevenson et al., 2006; Stevenson and James, 2009; Werner and Noppeney, 2010). A recent study found that the theta-band functional connectivity of older adults to the audiovisual dynamic hand-held tool was higher than that of younger adults (Ren et al., 2020). Dynamic hand tools, including the continuous process of early tool representation and late tool usage, are important for studying how older adults dynamically integrate visual and auditory information during this process. Therefore, this study used a dynamic hand-held tool as stimulation material to further explore the dynamic processing mechanism of audiovisual integration in older adults.

Previous research has found that stimulus intensity is one of the most important factors influencing audiovisual integration. Many behavioral studies involving younger adults have shown that low-intensity audiovisual stimuli can lead to more robust behavioral facilitation than high-intensity audiovisual stimuli (Corneil et al., 2002; Otto et al., 2013). Subsequent ERP studies using meaningless stimuli investigated the neurophysiological basis of the relationship between stimulus intensity and audiovisual integration. The results showed that after 40–60 ms of stimulus onset, audiovisual integration occurred only with a low-intensity stimulus in the left posterior and right anterior regions, but no audiovisual integration was observed in the medium- and high-intensity conditions (Senkowski et al., 2011). This phenomenon of enhanced audiovisual integration in low-intensity conditions is known as inverse effectiveness. However, a recent study using the same meaningless stimulus found that there was no difference in the audiovisual integration of older adults under different intensity conditions, indicating that older adults did not exhibit inverse effectiveness (Yang et al., 2021). For the complex speech stimulus, some studies found that at the whole-word level, the greater audiovisual integration in older adults than in younger adults under the intermediate stimulus intensity, but older adults reduced benefit at low stimulus intensity. In contrast, at the phoneme level, older adults showed similar audiovisual enhancement as younger adults when the stimulus intensity decreased (Stevenson et al., 2015). These results indicated that the inverse effectiveness was different between the younger and older adults across the different stimulus types. Some researchers used dynamic natural scenes as stimuli and found that dynamic high-intensity audiovisual stimuli are integrated through the direct exchange of information between the visual and auditory sensory cortex, while dynamic low-intensity audiovisual stimuli rely on the integration of input from the intraparietal sulcus (IPS), which may be related to the executive cortex network (Regenbogen et al., 2018). The dynamic hand-held tool was not only associated with motor execution but also associated with episodic memory. However, the effect of different intensities of the dynamic hand-held tool on audiovisual integration in older adults was unclear. Due to the instability of the inverse effectiveness in older adults, as well as the decline in executive function and episodic memory (Cepeda et al., 2001; Isingrini and Taconnat, 2008), it is necessary to further explore the effect of dynamic hand-held tools in different stimulus intensities on audiovisual integration in older adults.

The present study used dynamic hand-held tools to compare audiovisual integration between high- and low-intensity conditions to explore the dynamic processing mechanism of the audiovisual integration of motion-related tools in older and younger adults. We divided dynamic hand-held tools stimuli into high- and low-intensity stimuli and had the subjects perform a discrimination task in a random presentation of visual, auditory, and audiovisual stimuli. At the same time, we used ERPs to record the brain activity of subjects as they completed the task. Based on the above literature, it was hypothesized that stimulus intensity moderated and played a key role in the audiovisual integration of older and younger adults.

## Materials and Methods

### Participants

Twenty older adults (6 males, 14 females; mean age = 63.7 years, SD = 2.9 years) and 24 younger adults (11 males, 13 females; mean age = 21.4 years, SD = 2.6 years) participated in this experiment. All subjects had normal or corrected-to-normal vision and hearing and were free of chronic or neurological diseases through self-report. All subjects were right-handed. In addition, to exclude cognitive impairments that might affect the experimental results, older subjects were screened using the Simple Mental State Examination (MMSE). Subjects whose score was higher than 26 were considered normal (Bravo and Hébert, 1997). Due to recording EEG data, subjects were asked to keep their head and body as stable as possible during the experiment and to reduce blinking. One older participant withdrew from the experiment due to physical fatigue during the low-intensity block, and one older subject and four younger subjects were excluded due to excessive EEG artifacts resulting in fewer than 30 remaining segments. Finally, 18 older adults (6 males, 12 females; mean age = 63.0 years, SD = 2.9 years) and 20 younger adults (10 males, 10 females; mean age = 21.6 years, SD = 2.7 years) completed the entire experiment, and data were collected for further analysis. The average number of remaining epochs in older adults was 111.49 ± 21.83 (high-intensity condition: visual = 115.72 ± 25.76, auditory = 110.78 ± 29.74, audiovisual = 114.06 ± 27.31; low-intensity condition: visual = 111.44 ± 11.53, auditory = 107.88 ± 15.63, audiovisual = 108.31 ± 13.31). The average number of remaining epochs in younger adults was 96.91 ± 23.23 (high-intensity condition: visual = 101.28 ± 21.87, auditory = 94.89 ± 24.56, audiovisual = 97.67 ± 25.83; low-intensity condition: visual = 102.11 ± 20.35, auditory = 94.72 ± 23.60, audiovisual = 96.11 ± 23.13). All subjects were blinded to the purpose of this study and gave informed consent for the experiment. Each subject was paid a monetary reward after the experiment. The experiment had previously been approved by the ethics committee of Hubei University.

### Stimuli

Stimuli consisted of two dynamic hand-held tools (hammer and stick) that were recorded with a MiniDV Digital Camcorder (Sony, DCR-PC55). The video time is 800 ms, including the complete movement cycle and percussive sound of the hand-held tool. To separate the visual and auditory stimuli, Adobe Premiere CS6 was used to extract visual and auditory files from the original video. The visual stimuli were the videos acquired from the original video of 966 × 544 pixels and converted from color to grayscale. The auditory stimuli were the sound acquired at the original video of 32-bit audio with a sampling rate of 48 kHz and converted from stereo to mono. The intensity of the visual stimuli was regulated by modulating the contrast level, (max − min)/(max + min), with the high-intensity contrast being 90% and the low-intensity contrast being 10%. The intensity of the auditory stimuli was regulated by modulating the sound pressure level (SPL), with the high-intensity stimuli at 70 dB and the low-intensity stimuli at 50 dB. The selection of these methods was based on previous studies to ensure their effectiveness (Ren et al., 2020; Yang et al., 2021).

During the experiment, the stimuli were divided into target stimuli and standard stimuli. The visual target stimulus was the dynamic video of holding a wooden stick to hit the floor, and the visual standard stimulus was the dynamic video of holding a hammer hitting the floor. The auditory target stimulus was the sound of a wooden stick hitting the marble floor, and the auditory standard stimulus was the sound of the hammer hitting the marble floor. The audiovisual target stimulus was the combination of the dynamic video of holding a wooden stick to hit the floor and the sound of a wooden stick hitting the marble floor. The audiovisual standard stimulus was the combination of the dynamic video of holding a hammer hitting the floor and the sound of the hammer hitting the marble floor.

### Procedure and task

**Figure 1:**
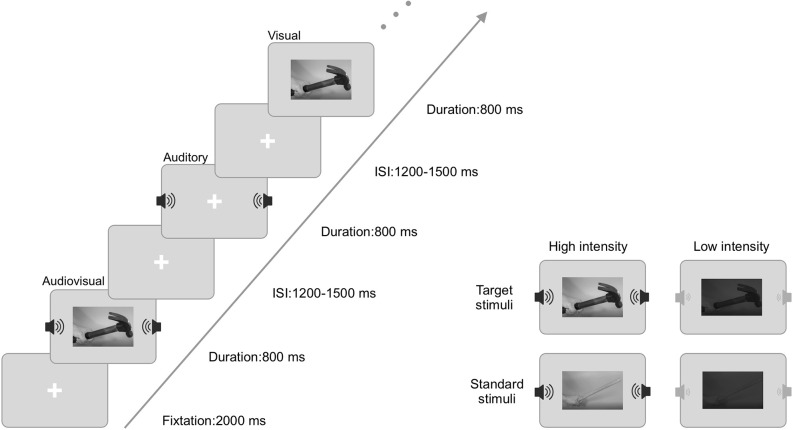
Schematic diagram of the experimental design.

The experimental procedure was carried out using E-prime 2.0 software. As shown in Figure 1, a 2,000 ms fixation point was presented at the beginning of each block. After the fixation point, target stimuli (visual, auditory, audiovisual) and standard stimuli (visual, auditory, audiovisual) were randomly presented for 800 ms. Then, a random trial interstimulus interval (ISI) was between 1,200 ms and 1,500 ms. Visual stimuli were presented using a Dell computer monitor at approximately 60 cm from the monitor. The visual stimulus had a vertical visual angle of 5° and a horizontal visual angle of 12°. The auditory stimuli were presented *via* speakers located on the left and right sides of the computer monitor.

The experiment included a high-intensity stimulus block and a low-intensity stimulus block. Each block contained 450 trials: 150 visual trials, 150 auditory trials, and 150 audiovisual trials, and each modality included 30 target trials and 120 standard stimulus trials. During the experiment, the laboratory room was dark and quiet. The participants were asked to fix their eyes on the screen and respond quickly and accurately to the target stimuli (left mouse button) but not to the standard stimuli. In the process of EEG recording, the participants were told to minimize blinking and keep their bodies and heads stable to avoid excessive artifacts. The duration of each block was approximately 16 min, and the subjects were allowed to rest every 4 min. High-intensity and low-intensity stimuli were administered in two blocks and randomly assigned between subjects.

### EEG recording and preprocessing

The EEG data were recorded with a 32-channel cap using the BrainAmp MR plus system and Brain recorder software. The resistance of reference and the other interelectrode impedances were set below 5 kΩ. Vertical and horizontal electrooculograms (EOGs) were recorded by two pairs of electrodes to monitor eye movements and eye blinks. The raw signals were digitized with a sampling frequency of 250 Hz.

All the data were stored digitally for offline analysis. EEG data preprocessing was conducted in Brain analysis software, including rereferencing to the bilateral mastoid electrodes (TP9 and TP10). The ERP data elicited by the standard stimuli were analyzed. Epochs were cut from 100 ms pre-stimuli to 400 ms post-stimulus onset for each trial. The original data were bandpass filtered from 0.01 to 60 Hz, and the time window between 100 ms and 0 ms before stimulus presentation was used as baseline correction. In addition to a ±100 μV artifact rejection criterion, EEG epochs containing eye blinks or other noise transients were removed based on a trial-by-trial inspection of the data. Then, ERP data were grand-averaged for each stimuli modality, followed by digital filtering with a bandpass filter of 0.1–30 Hz.

## Data Analysis

### Behavioral data analysis

The hit rate (HT) was the percentage of correct responses relative to the total number of target stimuli. The false alarm (FA) was the percentage of incorrect responses relative to the total number of standard stimuli. The response times (RTs) were calculated based on the responses that fell within the average period ±3 SD. The number of trails remaining under each condition accounted for more than 97.5% of the total number of trials. Then, a three-mixed factor analysis of variance (ANOVA) of age (younger, older), stimulus intensity (high, low), and modality (visual, auditory, audiovisual) was conducted. If ANOVA revealed a significant interaction effect, subsequent simple effect analysis was conducted separately for each factor.

### ERP analysis

The multisensory integration effect was measured by the different amplitudes between the AV and A + V. The effectiveness of this method is attested by a statistical hypothesis that the amplitudes evoked by the neural activities by audiovisual stimuli (AV) should be equivalent to the summation of the amplitudes evoked by the neural activities by unimodal stimuli plus (A + V). However, if the amplitude of ERP(AV) − [ERP(A) + ERP(V)] was significantly greater or less than zero, it can be inferred that audiovisual integration occurred (Giard and Peronnet, 1999; Senkowski et al., 2007).

To measure the audiovisual integration between the different situations, the statistical analysis was conducted in the following steps. First, pointwise running *t*-tests were used. The mean amplitudes of ERP(AV) − [ERP(A) + ERP(V)] were compared with 0 at each time point from 0 to 400 ms in each electrode. If 12 (24 ms) or more consecutive points were significant (criterion *p* < 0.050), we defined that audiovisual integration occurred. Based on the *t*-test analysis, the time course showed four audiovisual integration time windows (60–100 ms, 120–160 ms, 220–260 ms, 340–380 ms). Furthermore, the time course showed possible lateralization of audiovisual integration. In previous research, audiovisual integration might appear in the hemisphere of the brain (Molholm et al., 2002; Diaconescu et al., 2013). Therefore, based on the results of previous and present studies, the five regions of interest (ROIs) were set as left anterior (F3, FC5, FC1), right anterior (F4, FC6, FC2), central (C3, Cz, C4), left posterior (P3, CP5, CP1), and right posterior (P4, CP6, CP2). Subsequently, the wave amplitudes of the electrodes in each region were averaged for inclusion in subsequent data analysis. Second, a three-factor mixed-design ANOVA 2 (age: younger, older) × 2 (stimulus intensity: high, low) × 5 (ROI: left anterior, right anterior, central, left posterior, right posterior) was conducted for the selected time interval by using the amplitudes of ERP(AV) − [ERP(A) + ERP(V)]. Age was a between-subject factor, and stimuli intensity and ROI were within-subject factors. The SPSS version 26.0 software package was used for all statistical analyses and Greenhouse-Geisser corrections with corrected degrees of freedom.

## Results

### Behavioral results

The hit rate was analyzed with a 2 (age: younger adults, older adults) × 2 (stimulus intensity: high, low) × 3 (modality: visual, auditory, audiovisual) mixed-factors ANOVA. The analysis only revealed that the modality main effect was significant, *F*_(2,72)_ = 7.86, *p* = 0.002, $\eta_{p}^{2}$ = 0.18, with the hit rate of the audiovisual modality being higher than that of the visual, *p* = 0.013, and auditory modality, *p* < 0.001. The hit rate of the visual modality and auditory modality was not significantly different, *p* = 0.058. No significant interaction effect was observed for the hit rate, *ps* > 0.050. The detailed hit rates are shown in Table 1.

**Table 1:** Response times and hit rates for both the younger and older adults under the high-intensity and low-intensity conditions.

	**Low-intensity**			**High-intensity**		
	**Visual**	**Auditory**	**Audiovisual**	**Visual**	**Auditory**	**Audiovisual**
Older adults			
HT(%)	97 (6)	95 (6)	98 (3)	97 (3)	96 (8)	99 (3)
FA(%)	0.14 (0.32)	1.39 (2.93)	0.48 (0.60)	0.76 (2.36)	1.73 (2.08)	0.97 (1.81)
RT(ms)	655 (111)	673 (116)	575 (93)	590 (104)	662 (74)	562 (71)
Younger adults			
HT(%)	98 (2)	97 (5)	99 (2)	99 (2)	97 (4)	99 (3)
FA(%)	0.17 (0.34)	0.71 (1.10)	0.25 (0.48)	0.21 (0.37)	1.74 (1.51)	0.38 (0.63)
RT(ms)	521 (67)	584 (71)	502 (62)	543 (55)	573 (65)	468 (62)

Note. Response times were the median. Standard deviations are given in parentheses.

The false alarm was analyzed using a 2 (age: younger, older) × 2 (stimulus intensity: high, low) × 3 (modality: visual, auditory, audiovisual) mixed-factors ANOVA. The analysis revealed that the main effect of age was not significant, *F*_(1,36)_ = 2.82, *p* = 0.102, $\eta_{p}^{2}$ = 0.07. The main effect of stimulus intensity was significant, *F*_(1,36)_ = 4.29, *p* = 0.046, $\eta_{p}^{2}$ = 0.11, with the false alarm to the high-intensity stimulus being greater than that to the low-intensity stimulus. The modality main effect was significant, *F*_(2,72)_ = 10.63, $\eta_{p}^{2}$ = 0.38, *p* < 0.001, with the false alarm of the auditory modality being greater than that of the audiovisual modality, *p* < 0.001, and visual modality, *p* < 0.001. The false alarm of the audiovisual modality and visual modality were not significantly different, *p* = 0.303. No other significant interaction effect was observed for false alarm, *ps* > 0.050. The detailed false alarms are shown in Table 1.

The response time was analyzed using a 2 (age: younger, older) × 2 (stimulus intensity: high, low) × 3 (modality: visual, auditory, audiovisual) mixed-factors ANOVA. The analysis revealed that the main effect of age was significant, *F*_(1,36)_ = 12.96, *p* = 0.001, $\eta_{p}^{2}$ = 0.27, with the response time of older adults being longer than that of younger adults. The main effect of stimulus intensity was significant, *F*_(1,36)_ = 6.03, *p* = 0.019, $\eta_{p}^{2}$ = 0.14, with the response time to the high-intensity stimulus being shorter than that to the low-intensity stimulus. The modality main effect was significant, *F*_(2,72)_ = 99.31, $\eta_{p}^{2}$ = 0.73, *p* < 0.001, with the response time of the audiovisual modality being shorter than that of the visual modality, *p* < 0.001, and auditory modality, *p* < 0.001. Moreover, the response time of the visual modality was shorter than that of the auditory modality, *p* = 0.001 (AV < V < A). Additionally, there was a significant interaction between the group and modality, *F*_(2,72)_ = 4.14, $\eta_{p}^{2}$ = 0.10, *p* = 0.021. Further simple effect analysis showed that for all modalities, the response time of younger adults was shorter than that of older adults, *ps* ≤ 0.008. For both younger and older adults, the response to the audiovisual modality was the fastest, *ps* ≤ 0.016. No other significant interaction effect was observed for response time, *ps* > 0.050. The detailed response times are shown in Table 1.

### ERP results

#### Audiovisual integration at 60–100 ms

A three mixed-factor ANOVA revealed that the main effect of age was not significant, *F*_(1,36)_ = 0.35, *p* = 0.560, $\eta_{p}^{2}$ = 0.01. The main effect of stimulus intensity was marginally significant, *F*_(1,36)_ = 3.92, *p* = 0.055, $\eta_{p}^{2}$ = 0.10, with the amplitudes of the low-intensity stimulus being more negative than those of the high-intensity stimulus. The ROI main effect was also significant, *F*_(4,144)_ = 3.37, *p* = 0.029, $\eta_{p}^{2}$ = 0.09, with the amplitudes of the right anterior being more negative than those of the left anterior, central, and left posterior, *ps* < 0.040. The amplitudes of the central and right posterior regions were more negative than those of the left posterior region, *ps* < 0.033. A three-factor interaction effect was significant, *F*_(4,144)_ = 9.86, *p* < 0.001, $\eta_{p}^{2}$ = 0.22, and the stimulus intensity and age interaction effect were also significant, *F*_(1,36)_ = 11.92, *p* = 0.001, $\eta_{p}^{2}$ = 0.25.

**Figure 2:**
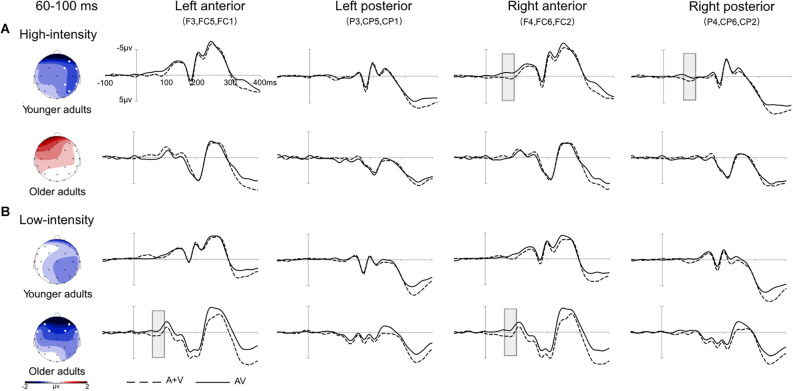
Grand-averaged event-related potentials and topography map of audiovisual integration in the time window of 60–100 ms. Grand-averaged event-related potentials included the left anterior (F3, FC5, FC1), left posterior (P3, CP5, CP1), right anterior (F4, FC6, FC2), and right posterior (P4, CP6, CP2) regions. The solid line is the event-related potentials of the audiovisual stimulus (AV), and the dotted line is the sum of the event-related potentials of visual and auditory stimuli (A + V). The areas where audiovisual integration increases are marked with gray squares. The topographic map shows the difference between AV and A + V. **(A)** Under the high-intensity condition, audiovisual integration was greater in the right anterior and right posterior regions in young adults than in older adults. **(B)** Under the low-intensity condition, audiovisual integration in the left anterior and right anterior regions was greater in older adults than in younger adults.

Further simple effect analysis indicated that in the high-intensity condition, the amplitudes of the younger adults were more negative than those of older adults in the left anterior, central, and right anterior directions, *ps* < 0.047. In the low-intensity condition, the amplitudes of the older adults were more negative than those of younger adults in the left and right anterior directions, *ps* < 0.017. For the younger adults, in the high-intensity condition, the amplitudes of the right anterior were more negative than those of the left anterior, central, and left posterior, *ps* < 0.035, and the amplitudes of the right posterior were marginally more negative than those of the left posterior, *p* = 0.056. In the low-intensity condition, the amplitudes of the right posterior were more negative than those of the left posterior, *p* = 0.028. However, the amplitudes of the high-intensity condition and low-intensity condition were not significantly different in all regions, *ps* > 0.050. For the older adults, in the high-intensity condition, the amplitudes of all regions were not significant, *ps* > 0.050. However, in the low-intensity condition, the amplitudes of the left and right anterior were more negative than those of the left and right posterior, *ps* < 0.036, and the amplitudes of the center were more negative than those of the left anterior, *p* = 0.014. Furthermore, the amplitudes of the low-intensity condition were more negative than those of the high-intensity condition in the left anterior, right anterior, central, and right posterior, *ps* < 0.005. Grand-averaged event-related potentials and topography maps of audiovisual integration in the time window of 60–100 ms are shown in Figure 2.

#### Audiovisual integration at 120–160 ms

A three mixed-factor ANOVA revealed that the main effect of age was not significant, *F*_(1,36)_ = 0.01, *p* = 0.945, $\eta_{p}^{2}$ = 0.01. The stimulus intensity main effect was significant, *F*_(1,36)_ = 4.97, *p* = 0.032, $\eta_{p}^{2}$ = 0.12, with the amplitudes of the low-intensity condition being more negative than those of the high-intensity condition. However, the main effect of ROI was not significant, *F*_(4,144)_ = 3.36, *p* = 0.052, $\eta_{p}^{2}$ = 0.09. A three-factor interaction effect was significant, *F*_(4,144)_ = 3.25, *p* = 0.005, $\eta_{p}^{2}$ = 0.08, and the age and intensity interaction effect were also significant, *F*_(1,36)_ = 4.33, *p* = 0.045, $\eta_{p}^{2}$ = 0.11.

Further simple effect analysis indicated that for the older adults, the amplitudes of the low-intensity condition were more negative than those of the high-intensity condition, *p* = 0.005. Furthermore, in the low-intensity condition, the amplitudes of the left and right anterior were more negative than those of the central, left posterior, and right posterior in older adults, *ps* < 0.044. No significant effect was observed for other conditions, *ps* > 0.050.

#### Audiovisual integration at 220–260 ms

A three mixed-factor ANOVA revealed that the main effect of age was not significant, *F*_(1,36)_ = 0.06, *p* = 0.805, $\eta_{p}^{2}$ = 0.01. The main effect of stimulus intensity was not significant, *F*_(1,36)_ = 1.90, *p* = 0.177, $\eta_{p}^{2}$ = 0.05. The main effect of ROI was also not significant, *F*_(4,144)_ = 3.03, *p* = 0.064, $\eta_{p}^{2}$ = 0.08. However, a three-way interaction effect was significant, *F*_(4,144)_ = 2.88, *p* = 0.025, $\eta_{p}^{2}$ = 0.07, and other interaction effects were not significant, *ps* > 0.050.

Further simple effect analysis indicated that for the older adults, the amplitudes of the low-intensity condition were more negative than those of the high-intensity condition, *p* = 0.019. In the low-intensity condition, the amplitudes of the right anterior were more negative than those of the left anterior and posterior, *ps* < 0.044. No significant effect was observed for other conditions, *ps* > 0.050.

#### Audiovisual integration at 340–380 ms

A three mixed-factor ANOVA revealed that the main effect of age was not significant, *F*_(1,36)_ = 0.01, *p* = 0.943, $\eta_{p}^{2}$ = 0.01. The main effect of stimulus intensity was not significant, *F*_(1,36)_ = 3.37, *p* = 0.075, $\eta_{p}^{2}$ = 0.09. The main effect of ROI was also not significant, *F*_(4,144)_ = 2.59, *p* = 0.074, $\eta_{p}^{2}$ = 0.07. However, the age and ROI interaction effect was significant, *F*_(4,144)_ = 5.69, *p* = 0.003, $\eta_{p}^{2}$ = 0.14. The other interaction effects were not significant, all *ps* > 0.050.

**Figure 3:**
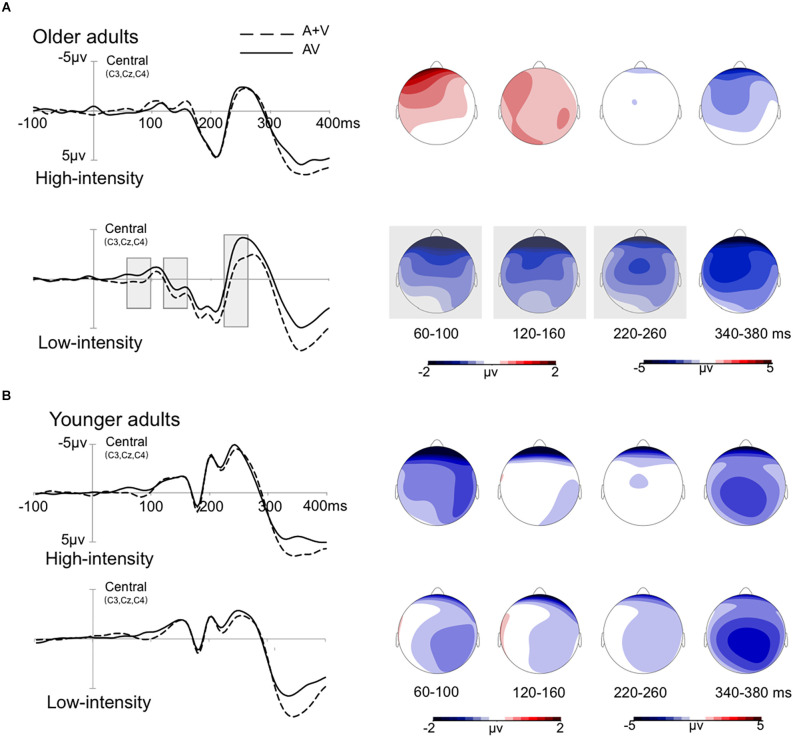
Grand-averaged event-related potentials and topography map of audiovisual integration in the time window of 60–100 ms, 120–160 ms, 220–260 ms, and 340–380 ms. The wave amplitude is the average of the wave amplitudes of the three electrodes C3, Cz, and C4. The solid line is the event-related potentials of the audiovisual stimulus (AV), and the dotted line is the sum of the event-related potentials of visual and auditory stimuli (A + V). The areas where audiovisual integration increases are marked with gray squares. The topographic map shows the difference between AV and A + V. **(A)** The amplitude of waves in older adults under high- and low-intensity conditions. **(B)** The amplitude of waves in the younger adults under high- and low-intensity conditions.

Further analysis shows that for the younger adults, the amplitude of the central region was more negative than that of the left anterior, *p* = 0.049. For older adults, the amplitudes of the left anterior, right anterior, and central regions were more negative than those of the left and right posterior regions, *ps* < 0.047. No significant effect was observed for other conditions, *ps* > 0.050. Grand-averaged event-related potentials and topography maps of audiovisual integration of older adults in the high- and low-intensity conditions are shown in Figure 3.

## Discussion

This study found that both older and younger adults responded faster and at higher hit rates to audiovisual stimuli than to visual and auditory stimuli. The response time of the older adults was significantly longer than that of the younger adults in each condition. ERPs revealed that during the early integration stage of 60–100 ms, in the low-intensity condition, audiovisual integration of the left anterior and right anterior regions was greater in older adults than in younger adults; however, in the high-intensity condition, audiovisual integration of the right anterior and right posterior regions was greater in younger adults than in older adults. In addition, audiovisual integration was greater in the low-intensity condition than in the high-intensity condition in older adults during the 60–100 ms, 120–160 ms, and 220–260 ms periods, showing inverse effectiveness.

### Aging effect of audiovisual integration

During the early integration stage of 60–100 ms, audiovisual integration of the left anterior and right anterior regions was greater in older adults than in younger adults under low-intensity conditions. Some studies on younger adults found that in the early stage of processing, audiovisual integration generally appears in the primary processing cortex, such as the occipital cortex (Yang et al., 2015). In contrast, the study found audiovisual integration in the posterior parietal and medial prefrontal cortices in older adults during the early stage of processing. Increased activity in these regions in older adults predicts faster detection of cross-modality stimuli, suggesting that the posterior parietal and medial prefrontal cortices may be the compensatory regions of audiovisual integration in older adults (Diaconescu et al., 2013). Furthermore, according to the hemisphere asymmetric reduction hypothesis of older adults (HAROLD), older adults tend to show more symmetrical activation than younger adults (Cabeza, 2002; Cabeza et al., 2018). In aging studies, activation of the bilateral prefrontal cortex is generally considered to be an adaptive and compensatory neural mechanism for older adults, which is likely to compensate for the functional impairment of other brain areas. For example, some studies have found that in attention and memory tasks, brain activity in the bilateral prefrontal and precuneus regions increases in older adults (Velanova et al., 2007; Patrick et al., 2009). This compensation mechanism is adapted to the aging of brain structure and function, suggesting that compensatory brain activation in older adults could help maintain or improve cognitive performance. Therefore, the enhancement of audiovisual integration for older adults could be interpreted due to the compensatory mechanism of the brain recruitment strategy. In addition, the brain recruitment strategy in older adults may reflect the involvement of higher cognitive functions during the early stage of integrated processing. According to the cognitive permeation hypothesis, when older adults cannot measure stimulus information effectively, they might use higher cognitive functions to aid in sensory processing (Lindenberger et al., 2000). Under low-intensity conditions, due to the decrease in the contrast of visual stimuli and the loudness of sound stimuli, older adults may allocate more cognitive resources to participate in the early integration stage, resulting in greater audiovisual integration. On the other hand, compared with young adults, older adults are less able to suppress cross-modal information and are more susceptible to information from different sensory modalities (de Dieuleveult et al., 2017). Hugenschmidt et al. (2009) found that under the audiovisual condition, even if older adults were required to selectively pay attention to the information of a certain visual or auditory modality, the facilitation effect of audiovisual integration would also appear. However, under the condition of selective attention, the facilitation effect of audiovisual integration did not appear in younger adults (Hugenschmidt et al., 2009). Thus, the decreased ability to suppress cross-modality information could also explain the greater audiovisual integration in older adults than younger adults.

In addition, the current results showed that audiovisual integration in the right anterior and right posterior regions was greater in young adults than in older adults under high-intensity conditions. Studies have found that younger adults usually activate the right hemisphere when integrating motor-related stimuli (Senkowski et al., 2007). Moreover, the ERP results revealed that the audiovisual integration of younger adults appeared in the right parietal-occipital cortex 46 ms after stimuli presentation (Molholm et al., 2002). However, older adults did not exhibit a lateralization effect, which may be due to the dedifferentiation of the brain activation patterns caused by aging. Some studies have found that the differentiation of unilateral specific processing of the brain disappears with age, resulting in a decrease in the accuracy of information transmission and the clarity of mental presentation (Li et al., 2001). Older adults experience a decline in sensory function, including decreased visual acuity and increased auditory thresholds (Brooks et al., 2018; Jayakody et al., 2018). The declines in sensory function can further affect cognitive abilities, including deterioration in motor speed and executive function in older adults (Roberts and Allen, 2016). Considering that the effectiveness of multisensory integration depends on the functions of the sensory organs and cognitive processes (Talsma et al., 2010), it seems reasonable to observe a decline in audiovisual integration in older adults.

### Inverse effectiveness of audiovisual integration for older adults

The results showed that the audiovisual integration of older adults was greater in low-intensity conditions than in high-intensity conditions during the 60–100 ms, 120–160 ms, and 220–260 ms periods. The results might be due to attention acting on audiovisual integration at early and late stages. The study found that audiovisual integration was greatest when the stimulus intensity was close to the sensory threshold (Cappe et al., 2010). It is possible that low-intensity stimuli might drive bottom-up attention, thereby enhancing audiovisual integration in the early stage (Van Zoest et al., 2004). Considering that stimulus intensity is reduced to approach the perceptual threshold of older adults, low-intensity stimuli might drive more attention in older adults during the early integration stage than in younger adults. Furthermore, subjects also actively allocated top-down attention to complete the task in the late stage (Talsma et al., 2010; Talsma, 2015). Considering the decline in executive function and reaction flexibility in older adults (Cepeda et al., 2001; Grady, 2012), older adults might allocate more attentional resources in the late integration stage to maintain performance. The parallel integration framework suggests that multisensory integration can occur in multiple stages and is dynamically modulated by attentional resources (Calvert and Thesen, 2004). For older adults, increasing attention to cross-modality information was a better strategy when one or more sensory sources become unreliable, which can be effective in helping older adults complete the task. Thus, the audiovisual integration of older adults under low-intensity stimuli might be modulated by the dynamics of attention. It can be observed at different stages that the audiovisual integration of older adults is greater in the low-intensity condition than in the high-intensity condition.

In addition, the dynamic hand-held tool used as the experimental material in this study has a certain special characteristic. The dynamic hand-held tool not only contains images of the movement of the arm and tool but also contains the experience when using the tool. Some studies have found that the brain regions that are active in observing a specific behavior closely match the brain regions that are active in performing the same behavior (Beauchamp and Martin, 2007; James et al., 2011). This mirror system reflects the actual experience of the individual in observing tool use. According to the sensory/motor system, individuals match the visual and auditory stimuli during tool use and store them in the perceptual system and in the motor system responsible for grasping and manipulating tools (Martin et al., 2000; Barsalou et al., 2003). Due to long-term life experience and practice, older adults have a strong connection between visual and auditory aspects when using tools. Furthermore, older adults maintain a better level of crystal intelligence related to the accumulation of experience. When the stimulus intensity decreases, the crystal intelligence and the richness of experience can compensate for the lack of information when perceiving the stimuli. Some studies further found that the connection between vision and auditory function obtained through practice influences perceptual judgment. Furthermore, the connection between vision and auditory stimuli is enhanced when stimulus uncertainty increases (Kafaligonul, 2018). Therefore, when the intensity of the stimuli intensity decreases, the robust connection between the visual and auditory senses might facilitate audiovisual integration in older adults.

In summary, there was an age-related dissociation between high- and low-intensity conditions with audiovisual integration of the dynamic hand-held tool stimulus. Older adults showed greater audiovisual integration in the lower intensity condition, which may be explained by the activation of compensatory mechanisms. However, the research still had some limitations. The presentation of sound stimuli only used a speaker, not the headphone, which might not completely shield outside sounds. Furthermore, we presented high-intensity and low-intensity stimuli between blocks in the current study, as sequential presentations might bring about fatigue effect and practice effect. If the subjects completed the high-intensity stimulation block first, the fatigue effect would make it more difficult for them to complete the low-intensity stimulation block. On the other hand, if subjects completed the low-intensity stimulation block first, there was a practice effect that made it easier for them to complete the high-intensity stimulation block. The stimulation block completed first would affect the stimulation block completed later, due to the fatigue effect and the practice effect, which might further affect the behavior response and neural activity of the subjects. Although the study carried out a balance among subjects, the effect of sequence effects could not be completely removed. Therefore, in subsequent studies, we would consider presenting the high- and low-intensity stimulus intermixed to reduce the influence of additional variables on the experimental results.

## Data Availability Statement

The raw data supporting the conclusions of this article will be made available by the authors, without undue reservation.

## Ethics Statement

The studies involving human participants were reviewed and approved by Hubei University. The patients/participants provided their written informed consent to participate in this study.

## Author Contributions

All authors contributed to the study conception and design. Material preparation and data collection were performed by AG, SL, and ZL. Data analysis was performed by XY, YR, and JL. The first draft of the manuscript was written by XY and WY. All authors commented on previous versions of the manuscript. All authors contributed to the article and approved the submitted version.
